# Supplementary material for: Glycolysis upregulation is neuroprotective as a compensatory mechanism in ALS
Source: eLife. 2019 Jun 10;8:e45114. doi: 10.7554/eLife.45114 (PMC6557627; doi:10.7554/eLife.45114)
Supplement: Supplementary file 1. — Altered metabolites in third instar larvae crossed with the motor neuron driver D42 GAL4 were measured using gas or liquid chromatography followed by mass spectrometry. Red and green colored cells indicate statistically significant changes (Pvalue <0.05) that are increased and decreased, respectively. Light red and light green colored cells indicate upward or downward trends, respectively (Pvalue <0.1). [file elife-45114-supp1.docx]

**Supplemental file 1. Summary of carbohydrate metabolites in TDP-43^WT^ and TDP-43^G298S^ compared to w^1118^ controls.** Altered metabolites in third instar larvae crossed with the motor neuron driver D42 GAL4 were measured using gas or liquid chromatography followed by mass spectrometry. Red and green colored cells indicate statistically significant changes (P_value_ < 0.05) that are increased and decreased, respectively. Light red and light green colored cells indicate upward or downward trends, respectively (P_value_ < 0.1).

|  | **Biochemical name** | **TDP^WT^**  **control** | **P_value_** | **TDP^G298S^**  **control** | **P_value_** |
| --- | --- | --- | --- | --- | --- |
| Glycolysis, Gluconeogenesis and Pyruvate Metabolism | glucose | **0.85** | 0.0003 | 0.95 | 0.2562 |
|  | glucose-6-phosphate (G6P) | 1.13 | 0.697 | 1.37 | 0.1516 |
|  | glucose 1-phosphate | 0.83 | 0.4723 | 0.87 | 0.5666 |
|  | fructose-6-phosphate | 1.29 | 0.5165 | 1.35 | 0.235 |
|  | 2-phosphoglycerate | 0.77 | 0.9454 | 1.05 | 0.5316 |
|  | 3-phosphoglycerate | 1.03 | 0.9916 | 0.98 | 0.5479 |
|  | phosphoenolpyruvate (PEP) | **3.29** | 0.0343 | **5.35** | 0.0016 |
|  | pyruvate | **1.67** | 0.0072 | **1.6** | 0.0089 |
|  | lactate | 0.9 | 0.2946 | 0.92 | 0.4443 |
|  | glycerate | 1.05 | 0.5279 | 0.84 | 0.6078 |
| Pentose Phosphate Pathway | 6-phosphogluconate | **0.62** | 0.0174 | 1.1 | 0.7614 |
|  | sedoheptulose-7-phosphate | 1.16 | 0.5718 | **2.38** | 0.0084 |
|  | ribulose/xylulose 5-phosphate | 0.79 | 0.211 | **0.58** | 0.0107 |
| Pentose Metabolism | ribulose | 1.13 | 0.1944 | 0.91 | 0.8221 |
|  | ribose | **1.3** | 0.0543 | 0.9 | 0.7074 |
|  | ribitol | **0.61** | 0.0433 | **0.58** | 0.0332 |
|  | ribonate | 0.95 | 0.6298 | 1.03 | 0.7813 |
|  | xylonate | **0.73** | 0.0615 | 1.11 | 0.7787 |
|  | xylose | 0.9 | 0.6029 | 0.75 | 0.171 |
|  | xylitol | 0.89 | 0.1002 | **0.85** | 0.0266 |
|  | threitol | 1.4 | 0.4369 | **2.18** | 0.0355 |
|  | arabitol | 1.09 | 0.5878 | 1.3 | 0.194 |
| Glycogen Metabolism | maltopentaose | 0.86 | 0.4789 | 1.23 | 0.3532 |
|  | maltotetraose | 0.95 | 0.8841 | 0.98 | 0.8168 |
|  | maltotriose | 1.02 | 0.9606 | 0.95 | 0.7228 |
|  | maltose | **0.85** | 0.0094 | 0.97 | 0.6114 |
|  | isomaltose | 0.83 | 0.2842 | 0.91 | 0.6496 |
| Disaccharides and Oligosaccaharides | trehalose | 1.17 | 0.1078 | 1.04 | 0.6138 |
| Fructose, Mannose and Galactive Metabolism | fructose | 1.1 | 0.4677 | 1.05 | 0.7955 |
|  | sorbose | 1.16 | 0.3647 | 1.11 | 0.4997 |
|  | sorbitol | **0.8** | 0.0935 | 0.81 | 0.1236 |
|  | mannose | 0.84 | 0.297 | 0.98 | 0.9304 |
|  | mannose-6-phosphate | 1.15 | 0.6242 | 1.38 | 0.1272 |
|  | galactose 1-phosphate | 0.78 | 0.3283 | 0.92 | 0.82 |
|  | galactonate | **0.76** | 0.0366 | 0.95 | 0.8452 |
| Nucleotide Sugar | UDP-glucuronate | 0.82 | 0.4406 | 0.85 | 0.5114 |
|  | UDP-N-acetylglucosamine | **0.76** | 0.0224 | 0.86 | 0.2222 |
| Aminosugar Metabolism | glucosamine-6-phosphate | 0.94 | 0.911 | 1.33 | 0.1329 |
|  | glucuronate | **0.83** | 0.007 | **0.6** | 1.18E-09 |
|  | N-acetylglucosamine | **0.76** | 0.0519 | 0.8 | 0.1516 |
|  | N-acetylglucosamine 6-phosphate | **0.68** | 2.24E-05 | **0.84** | 0.0424 |
