# Supplementary material for: Glycolysis upregulation is neuroprotective as a compensatory mechanism in ALS
Source: eLife. 2019 Jun 10;8:e45114. doi: 10.7554/eLife.45114 (PMC6557627; doi:10.7554/eLife.45114)
Supplement: Supplementary file 2. [file elife-45114-supp2.docx]

| **Group/ID** | **Race** | **Gender** | **Age** | **PMI (hrs)** |
| --- | --- | --- | --- | --- |
| CON 1 | W | M | 22 | 6 |
| CON 2 | W | F | 71 | 5 |
| CON 3 | - | M | 72 | 7 |
| CON 4 | - | F | 57 | 32 |
| CON 6 | W | F | 53 | 4 |
| CON 7 | W | F | 51 | 5 |
| CON 8 | W | F | 57 | 11 |
| CON 9 | W | M | 76 | 13 |
| ALS 1 | W | F | 68 | 1 |
| ALS 2 | W | M | 69 | 10 |
| ALS 3 | W | F | 73 | 11 |
| ALS 4 | W | M | 53 | - |
| ALS 5 | W | F | 62 | 4 |
| ALS 6 | W | M | 53 | 5 |
| ALS 7 | W | F | 76 | 6 |
| ALS 8 | W | F | 63 | 5 |
| ALS 9 | W | M | 67 | 8 |

**Supplemental file 2.** Summary of demographic information for patient samples used to quantify PFKP, PFKM and G6PD.

PMI = post-mortem interval; COM = Non-neurologic disease control
