# Supplementary material for: Glycolysis upregulation is neuroprotective as a compensatory mechanism in ALS
Source: eLife. 2019 Jun 10;8:e45114. doi: 10.7554/eLife.45114 (PMC6557627; doi:10.7554/eLife.45114)
Supplement: Supplementary file 3. — Patient cell lines used for qPCR analysis are shown. [file elife-45114-supp3.docx]

**Supplemental file 3.** Summary of iPSC MNs used to quantify PFKP, PFKM and G6PD.

Cell lines used for qPCR analysis are shown.

| **Patient line number** | **Passage number** | **Days in culture** |
| --- | --- | --- |
| ALS 1 | 39 | 69 |
| ALS 1 | 35 | 70 |
| ALS 3 | 36 | 77 |
| Control 1 | 19 | 73 |
| Control 2 | 28 | 74 |
| Control 3 | 37 | 71 |
| Control 4 | 21 | 70 |
